# Constructal Design of Elliptical Cylinders with Heat Generating for Entropy Generation Minimization

**DOI:** 10.3390/e22060651

**Published:** 2020-06-12

**Authors:** Rong Wang, Zhihui Xie, Yong Yin, Lingen Chen

**Affiliations:** 1 College of Power Engineering, Naval University of Engineering, Wuhan 430033, China; hjgcdxwangrong@163.com; 2 Institute of Thermal Science and Power Engineering, Wuhan Institute of Technology, Wuhan 430205, China; yinyongwit@163.com; 3 School of Mechanical & Electrical Engineering, Wuhan Institute of Technology, Wuhan 430205, China

**Keywords:** constructal theory, entropy generation minimization, electronics cooling, discrete elliptical cylinder, heat source, generalized thermodynamic optimization

## Abstract

A heat dissipation model of discrete elliptical cylinders with heat generation on a thermal conduction pedestal cooled by forced convection is established. Constructal design is conducted numerically by taking the distributions of thermal conductivity and heat generating intensity as design variables, the dimensionless entropy generation rate (DEGR) as performance indicator. The optimal designs for discrete elliptical cylinders with heat generating are obtained respectively, i.e., there are optimal distributions of heat generating intensity with its fixed total amount of heat sources, and there are optimal distributions of thermal conductivity with its fixed total amount of heat sources. These optimums for minimum DEGRs are different at different Reynolds numbers of airflow. The heat generating intensity can be decreased one by one appropriately in the fluid flow direction to achieve the best effect. When the Reynolds number of airflow is smaller, the thermal conductivity of heat source can be increased one by one appropriately in the fluid flow direction to achieve the best effect; when the Reynolds number of airflow is larger, the thermal conductivity of each heat source should be equalized to achieve the best effect. The results can give thermal design guidelines for the practical heat generating devices with different materials and heat generating intensities.

## 1. Introduction

Electronic manufacture technology has developed quickly, and as electronic devices and equipment have been continually and highly miniaturized and integrated, the power per unit volume of the devices has increased continually, which has made their heat dissipation problems increasingly prominent. Research aiming to optimize electronic device and heat sink designs to enhance their heat dissipation has attracted the interest of many scholars [1,2]. In recent years, extensive and in-depth investigations for heat transfer optimization in the light of constructal theory [3,4,5,6,7,8,9,10,11,12,13,14,15,16,17,18,19,20,21,22,23,24] and entropy generation minimization [25,26,27,28,29,30,31,32,33,34,35,36,37,38,39,40,41,42,43,44,45,46,47,48,49,50,51,52,53,54,55,56,57,58,59,60] have flourished, which are beneficial for technologic development of electronics cooling.

Bejan, who investigated the universal physical mechanism of various natural dendritic structures [4,5,6,9,10,11,12,13,14,15,16,17,18,19,20,21,22,24], found the constructal law and proposed constructal theory from analyzing the formation and evolution of street network configurations in major cities around the world as an example in 1996 [3]. Constructal theory was firstly applied to design an optimal network of high conductivity material for electronics cooling, and the constructal law was found correspondingly [4]. The constructal law [4] stated that, “For a finite-size flow system to persist in time (to live), its configuration must change in time such that it provides easier and easier access to its currents.” For everything in nature, society and engineering, the configuration evolution in time follows this deterministic physics law [3,4,5,6,7,8,9,10,11,12,13,14,15,16,17,18,19,20,21,22,23,24]. Constructal theory not only accounts for and predicts the flow pattern/configuration evolution in nature and society, but also provides theoretical guidelines for the flow pattern/configuration design in various disciplines. The statement of constructal law can be further simplified as “the structures of matters come from their optimal performances [6].” The purpose of constructal design is to seek the optimal configuration with the best distribution of imperfections, so it was also named as a new philosophy of geometry.

For heat transfer optimization, the studies following constructal law can be classified as single-objective optimization, multi-objective optimization and multi-disciplinary optimization by optimization objectives; and these also can be classified as heat conduction optimization, convective heat and mass transfer optimization and various device and component optimization by study objects, and so on [4,5,6,9,10,11,12,13,14,15,16,17,18,19,20,21,22,24]. It has also been applied to the designs of cavities and fin heat sinks [24,61,62,63], channel heat sinks [64,65], heat exchangers [66,67,68,69], nonuniform heat generating units [70,71], evaporators [72,73,74], boiler superheater [75], tubular arrangements [76,77], dual-pressure turbine [78,79], etc., in recent years. For the constructal design in engineering, heat sources [80,81,82,83,84,85,86,87,88,89,90,91,92] is one of important and interesting research hotspots.

Da Silva and Gosselin [80] pointed out that increasing the cooling channel number doesn’t necessarily enhance the heat dissipation by investigating the constructal design of square components with heat generation in cooling channels. Gong et al. [81,82,83] investigated the constructal designs for two types of cylinders with heat generating which surrounded with a fin under the conditions of natural cooling, forced cooling and uniform heat transfer coefficient, respectively. Jassim and Muzychka [84] indicated that the non-uniform distribution of heat sources had better heat dissipation performance than that of uniform distribution. Tye-Gingra et al. [85] optimized the initial phase of heat flux density’s function and position of heat source by establishing a heat source model with sinusoidal variation over time of heat generating intensity. Shi and Dong [86] explored the constructal layout of discrete components with heat generating subjected to forced laminar flow. Fan et al. [87] calculated the heat dissipation performance with single and discrete cylindrical heat sources numerically and the results showed that the performances of multi-scale geometry and non-equal distribution of heat generating intensity were better than those of the single-scale geometry and equal distribution of heat generating intensity. Wang et al. [88] carried out the constructal design for several heat source models including discrete stationary and rotating cylinders, singe and discrete elliptical tables. Sarper et al. [89] investigated the constructal design for discrete multi-scale length heat sources in vertical ducts. In addition, Chen et al. [90], Aslan et al. [91] and Armaghani et al. [92] investigated the heat source layout designs.

Practical processes, such as fluid flow with finite pressure difference and heat transfer with finite temperature difference, are non-equilibrium irreversible processes from the thermodynamic point of view. Bejan [25,26] first derived the corresponding entropy generation rate (EGR) formula for fluid flow with finite pressure difference and heat transfer with finite temperature difference, and proposed the principle of entropy generation minimization (EGM). Many scholars [27,28,29,30,31,32,33,34,35,36,37,38,39,40,41,42,43,44,45,46] have performed extensive and in-depth researches on various processes [32,42], cycles [28,29,36,37], devices [30,31,35,40] and systems [44,45,46] based on EGM. Recently, various fluid flows such as Newtonian flow [93], carbon nanotube flow [94] and Darcy-Forchheimer nanofluid flow [95], microchannel heat sinks [96], heat exchangers [97,98,99], and so on, have been investigated with EGM. The conventional metrics, such as the heat transfer enhancement and pressure drop, evaluate convective heat transfer from the view point of the first law of thermodynamics, while the entropy generation analysis is from the view point of the second law of thermodynamics by uniformly characterizing the irreversibility of heat transfer and fluid friction. The purpose of entropy generation minimization is to seek the minimization of thermodynamic irreversibility which is characterized and quantified by the indicators such as entropy generation rate, entropy generation number, Bejan number, and so on [44,45,46].

From the references mentioned above, although the constructal design and entropy generation minimization have respective theoretical connotations, core positions and optimization purposes, one can see that there are internal physical relations between optimal configuration and entropy generation minimization for various heat and mass transfer processes and systems [6,9,10,13,14,18,19,22,24,31,34], and there is a very interesting research content in thermal design employing the method that combining constructal theory with entropy generation minimization for heat source optimization [87,88]. The heat sources mentioned in [80,81,82,83,84,85,86,87,88,89,90,91,92] were usually cylinders, square columns, elliptical tables, embedded plate structures, and so on. Based on the above combining method, this paper will establish a 3-D heat dissipation model of discrete elliptical cylinders with heat generating, and design heat generating intensity distribution and thermal conductivity distribution to reach the minimum total DEGR of system. This method herein can be adapted to the thermal design requirements of heat sources with different cross-section shapes in practical applications, and especially help to provide theoretical guidelines for the optimization of practical electronic components with different materials and heat generating intensities.

## 2. Heat Source Model and Numerical Method

### 2.1. Geometric Model

**Figure 1:**
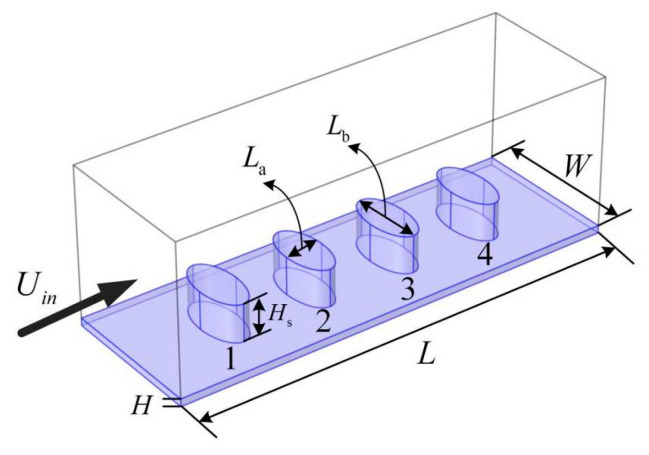
Geometric model of heat sources.

Figure 1 gives a geometric model of discrete elliptical cylinders with heat generating on a thermal conduction pedestal with forced convection in a rectangular channel. The length (*L*), width (*W*) and thickness (*H*) of thermal conduction pedestal is 60, 20 and 1 mm, respectively. Four equal-size discrete heat sources (numbered 1, 2, 3, and 4 from left to right) are evenly arranged along the central axis of pedestal, wherein the ellipse short axis (*L*_a_) of the heat source bottom surface is 2.5 mm, the ellipse long axis (*L*_b_) is 5 mm, and the height *H*_s_ of the heat source is 5 mm. The elliptical cylinders can only be arranged in line to meet some multi-disciplinary requirements for manufacture and performance in a practical engineering case.

### 2.2. Heat Transfer Model

The reference material of heat conductive pedestal is silicon (*c*_b_ is 700 J·kg^−1^·K^−1^, *ρ*_b_ is 2329 kg·m^3^, and *λ*_b_ is 130 W·m^−1^·K^−1^), the reference material of heat source is copper (*c*_s_ is 385 J·kg^−1^·K^−1^, *ρ*_b_ is 8960 kg·m^3^, *λ*_s_ is 400 W·m^−1^·K^−1^). The cooling medium through the rectangular channel is clean air, and the variable properties and viscosity dissipation of airflow are considered. The contact surfaces between airflow and channel wall, as well as that between airflow and heat source are all set with non-slip boundaries. The outer wall surfaces of channel and the bottom surface of pedestal are all set with adiabatic boundary conditions. The end faces at inlet and exit of the channel are open boundaries, and the ambient air temperature is set as *T*_in_ (293.15 K). The range of Prandtl number of air is 0.938–0.973. The forced airflow is a compressible steady-state turbulent flow, and the inlet airflow is perpendicular to the inlet end face.

The total EGR [25,26,44] of overall heat transfer process is generally clarified as the EGR of solid section and the EGR of fluid section, i.e.,:

(1) $$S_{g,\phi }=S_{\mathrm{solid}}+S_{\mathrm{fluid}}$$

The EGR of solid section [25,26,44] is:

(2) $$S_{\mathrm{solid}}=-\int_{V}\frac{1}{T^{2}}q\cdot \nabla TdV$$

The EGR of fluid section [25,26,44] is:

(3) $$S_{\mathrm{fluid}}=\int_{V}\left(-\frac{1}{T^{2}}q\cdot \nabla T+\frac{\mu }{T}\Phi \right)dV$$

where *q* (W·m^−2^) is the heat flux vector and Φ is a viscous dissipation function per unit volume. In the brackets of the right side of Equation (3), the first term is the EGR of heat transfer, and the second term is the EGR of fluid viscous dissipation. 

According to Equations (1)–(3), the total EGR of system is:

(4) $$S_{g,\phi }=-\int_{V_{s}}\frac{1}{T^{2}}q_{s}\cdot \nabla TdV-\int_{V_{b}}\frac{1}{T^{2}}q_{b}\cdot \nabla TdV+\int_{V_{f}}\left(-\frac{1}{T^{2}}q_{f}\cdot \nabla T+\frac{\mu }{T}\Phi \right)dV$$

where $q_{s}$, $q_{b}$ and $q_{f}$ are the heat flux vectors of heat sources, pedestal and fluid, respectively. *V*_s_, *V*_b_ and *V*_f_ are the volumes of the heat sources, pedestal and fluid, respectively.

According to Equation (4), the dimensionless entropy generation rate (DEGR) of system can be defined as:

(5) $${\tilde{S}}_{g,\phi }=\frac{S_{g,\phi }\cdot T_{\mathrm{in}}}{P_{t}}$$

The energy equation for solid pedestal with steady-state heat conduction and constant properties is:

(6) $$\nabla^{2}T=0$$

The energy equation for heat sources with steady-state heat conduction and constant properties is:

(7) $$\nabla^{2}T+\frac{q^{\prime \prime }}{\lambda_{s}}=0$$

Under forced convection condition, the governing equations of continuous fluid flow, momentum transfer, energy transfer and turbulence characteristics are as follows:

(8) ∇(ρU)=0

(9) $$\begin{array}{r} \rho \left(U\cdot \nabla \right)U=\nabla \cdot [-pI+(\mu +\mu_{T})(\nabla U+{\left(\nabla U\right)}^{T})\\ -\frac{2}{3}(\mu +\mu_{T})(\nabla \cdot U)I-\frac{2}{3}\rho kI]+F \end{array}$$

(10) $$\rho c_{f}U\cdot \nabla T+\nabla \cdot q=Q$$

(11) $$\rho (U\cdot \nabla )k=\nabla \cdot [(\mu +\frac{\mu_{T}}{\sigma_{k}})\nabla k]+P_{k}-\rho \varepsilon$$

(12) $$\rho (U\cdot \nabla )\varepsilon =\nabla \cdot [(\mu +\frac{\mu_{T}}{\sigma_{\varepsilon }})\nabla \varepsilon ]+C_{\varepsilon 1}\frac{\varepsilon }{k}P_{k}-C_{\varepsilon 2}\rho \frac{\varepsilon^{2}}{k}$$

(13) $$\mu_{T}=\rho C_{\mu }\frac{k^{2}}{\varepsilon }$$

(14) $$P_{k}=\mu_{T}[\nabla U:(\nabla U+{\left(\nabla U\right)}^{T})-\frac{2}{3}{\left(\nabla \cdot U\right)}^{2}]-\frac{2}{3}\rho k\nabla \cdot U$$

where the parameters with pulsation are all time-averaged. The empirical constants *C*_ε1_, *C*_ε2_, *C*_μ_, *σ*_k_ and *σ*_ε_ are 1.44, 1.92, 0.09 and 1.3, respectively.

The performance indicator for optimization is the total DEGR of system. The smaller the total DEGR of system, the better the thermodynamic performance of system. 

### 2.3. Numerical Method

The governing equations and boundary conditions of the heat dissipation model are solved by the finite element calculation method [100]. The tetrahedral meshing is performed in solid and fluid regions of the model, respectively. For reducing calculation deviations, the grid independence is tested. In the test case, the heat generating intensities (*q*” = 1.52 × 10^7^ W·m^−3^) and the thermal conductivities (*λ*_s_ = 200 W·m^−^^1^·K^−1^) of four heat sources are equal, and the inlet Reynolds number is set as 5000. There are three types of meshing with different grid numbers for computations, which are 12047, 52054 and 71006. The total DEGRs of system are 0.0170770, 0.0170654 and 0.0170732, and the relative errors are 0.068% and 0.046%, respectively. In order to balance the computation accuracy and efficiency, this paper uses the meshing criterion corresponding to 52054 grids for the following calculations. The general default convergence criteria for the continuity, momentum as well as energy equations are employed [100].

Reynolds Averaged Navier-Stokes (RANS) method is used to perform high-fidelity turbulence simulation calculations by Comsol Multiphysics. To further assess the accuracy of the computational model in this paper, 3-D models of cylindrical heat sources surrounded by fins with different dimensionless height $\tilde{H}$ and ratio *b* (the center-to-center distance of the fin and heat source to the radius of fin) in [82] are built for comparative calculations. The numerical results about dimensionless hot spot temperature $\tilde{T}$ are listed in Table 1.

For the same $\tilde{H}$ and *b*, the maximum difference of $\tilde{T}$ is only 1.27%, which means that the results herein agree well with the calculation results in [82]. The model of discrete elliptical cylinders with heat generating in this paper is established by the same method, so the effectiveness of the simulation method is verified.

## 3. Results and Analyses

Discrete electronic components with fixed positions always have various heat generating intensities and are made of different materials. Under forced convection conditions, the thermal conductivity distribution and the heat generating intensity distribution are chosen as design variables with the fixed total thermal conductivity and the fixed total heat generating intensity of heat sources, respectively. The constructal design of discrete elliptical cylinders with heat generating is conducted by taking the total dimensionless entropy generation rate minimization as performance indicator.

### 3.1. Effects of Heat Generating Intensity on Heat Dissipation Performance

The heat generating intensities from top to bottom are set as $q_{1}^{\prime \prime }$, $q_{2}^{\prime \prime }$, $q_{3}^{\prime \prime }$, and $q_{4}^{\prime \prime }$, respectively. The distribution is expressed as:

(15) $$q_{2}^{\prime \prime }-q_{1}^{\prime \prime }=q_{3}^{\prime \prime }-q_{2}^{\prime \prime }=q_{4}^{\prime \prime }-q_{3}^{\prime \prime }=\Delta q^{\prime \prime }$$

where $\Delta q^{\prime \prime }\;$is the difference of heat generating intensity.

The total heat generating intensity of heat sources is fixed, the total heating rate of the heat sources is *P*_t_ (48 W), so the heat generating intensity of each heat source is:

(16) $${q^{\prime \prime }}_{i}=15.2\times 10^{6}+\left(i-2.5\right)\Delta q^{\prime \prime }$$

**Figure 2:**
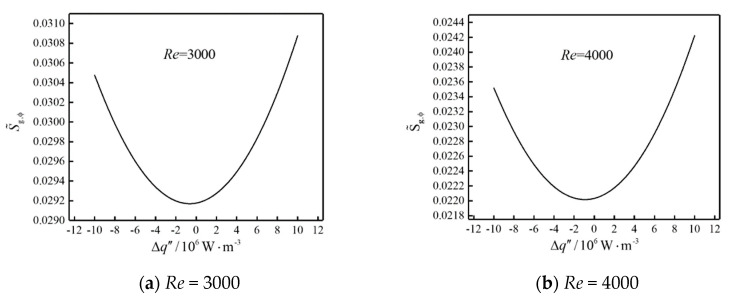

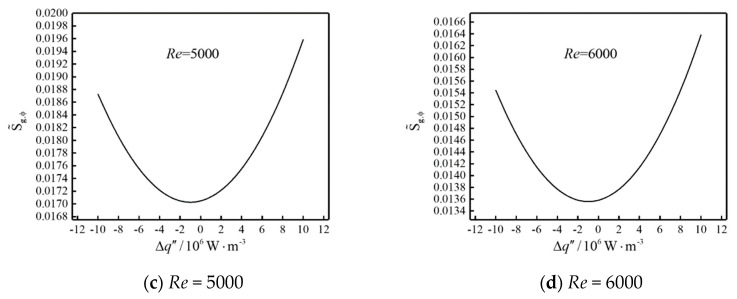
The influence of $\Delta q^{\prime \prime }$ on ${\tilde{\;S}}_{g,\phi }$.

The influence of $\Delta q^{\prime \prime }$ on ${\tilde{S}}_{g,\phi }$ of the system is shown in Figure 2 with the fixed equal thermal conductivity (400 W·m^−^^1^·K^−1^) for each heat source. The results show that the higher the Reynolds number (*Re*), the smaller the system temperature gradient, which makes the total DEGR (${\tilde{S}}_{g,\phi }$) reduce with the increase of airflow *Re* for specified heat generating intensity distribution. At the same *Re*, as the $\Delta q^{\prime \prime }$ increases, the ${\tilde{S}}_{g,\phi }$ decreases firstly and then increases, the optimal difference ($\Delta q_{\mathrm{opt}}^{\prime \prime }$) of heat generating intensity makes ${\tilde{S}}_{g,\phi }$ minimum. ${\tilde{S}}_{g,\phi }$ at $\Delta q^{\prime \prime }=-10\times 10^{6}$ W·m^−^^1^·K^−1^ is larger than that at $\Delta q^{\prime \prime }=1010^{6}$ W·m^−3^, which means that the heat generating intensity is preferably distributed from large to small in the airflow direction, the device with higher heat generating intensity should be arranged near the channel inlet. Furthermore, numerical results listed in Table 2 show that optimal differences ($\Delta q_{\mathrm{opt}}^{\prime \prime }\;s$) for the same optimization objective are different under different fluid flow conditions. From Table 2, all $\Delta q_{\mathrm{opt}}^{\prime \prime }\;s$ are a little bit less than 0. There is a very thin boundary layer improves the heat transfer at the upstream body and thus reduces temperature gradients. The three downstream bodies are affected by the weakening of upstream body. That is the upstream body could tolerate a higher heat flow rate than its downstream fellows. When $\Delta q^{\prime \prime }$ is close to the $\Delta q_{\mathrm{opt}}^{\prime \prime }\;$vaue, the cooling capacity of air on the part with high heat load is also strong, and it is relatively easy to avoid insufficient cooling of the part with high heat load. When $\Delta q^{\prime \prime }$ is less than $\Delta q_{\mathrm{opt}}^{\prime \prime }\;,{\tilde{\;S}}_{g,\phi }$ increases when $\Delta q^{\prime \prime }$ decreases. This is because the effect of heat generating intensity distribution on temperature gradient is more significant compared with that of boundary layer.

**Figure 3:**
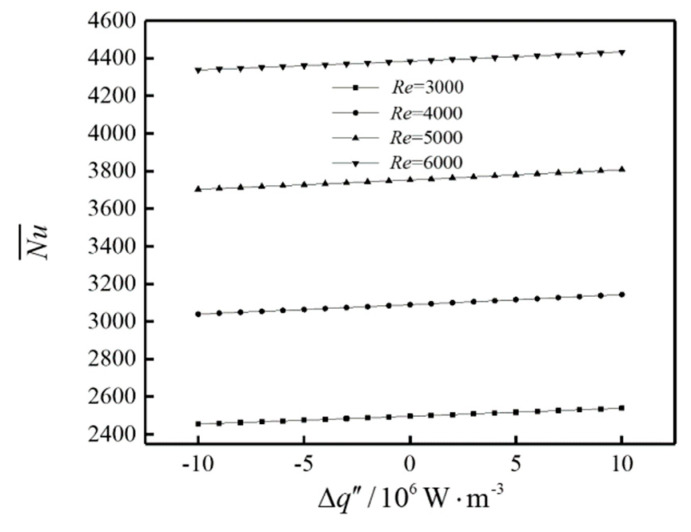
The influence of $\Delta q^{\prime \prime }$ on $\bar{Nu}$.

The influence of $\Delta q^{\prime \prime }$ on the average Nusselt number ($\bar{Nu}$) of the system is shown in Figure 3. It can be seen from Figure 3 that $\bar{Nu}$ increases with the increases of the *Re* and $\Delta q^{\prime \prime }$.

**Figure 4:**
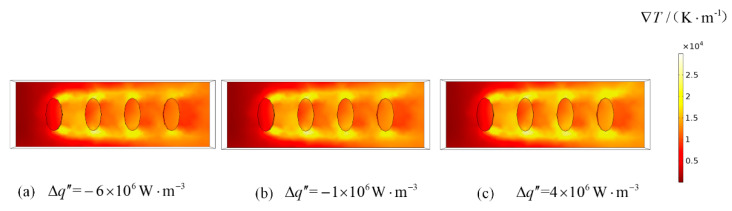
The color maps of temperature gradient distribution on the upper surfaces of discrete elliptical cylinders with heat generating and thermal conduction pedestal (*Re* = 5000).

**Figure 5:**
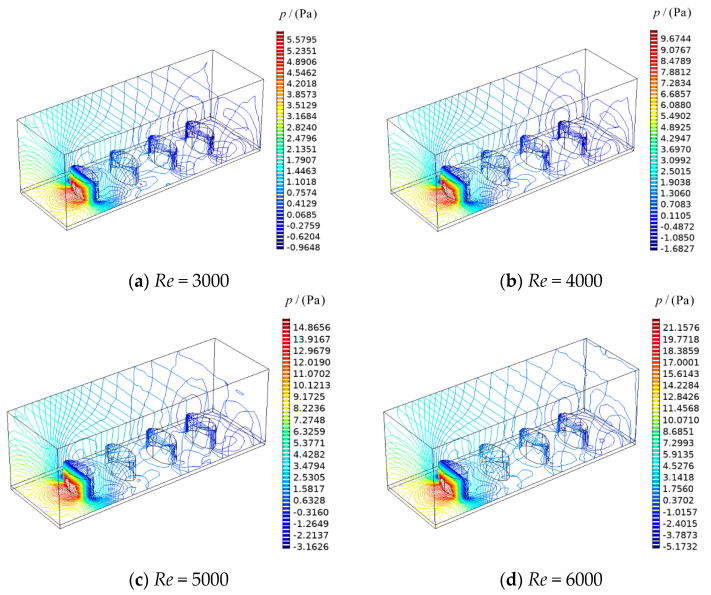
Contour plot for pressure in channel ($\Delta q^{\prime \prime }$ = 0 W·m^−3^).

The color maps of the temperature gradient distribution on upper surfaces of heat sources and thermal conduction pedestal at *Re* = 5000 are shown in Figure 4. From the figure, as the difference of heat generating intensity increases, the heat generating intensity in the flow direction which is from large to small changes to the distribution which is small to large, the temperature gradient distribution gradually changes from decreasing in flow direction to distributing uniformly around the heat sources. From Figure 4a, although both the heat generating intensity and the temperature gradient decrease in flow direction, the heat generating intensity at channel inlet is relatively larger which makes the heat source near inlet is not well cooled with $\Delta q^{\prime \prime }$ = −6 × 10^6^ W·m^−3^ the total EGR of system is higher. From Figure 4b, the temperature gradients around the heat sources gradually decrease in flow direction with $\Delta q^{\prime \prime }$ = −1 × 10^6^ W·m^−3^. In this case, the cooling of each heat source is optimally balanced, so that the total DEGR of system is the lowest. From Figure 4c, the temperature gradients around the four heat sources are substantially equal, but the heat generating intensity gradually increases one by one in flow direction with $\Delta q^{\prime \prime }$ = 4 × 10^6^ W·m^−3^, which leads to poor cooling of the heat source near channel outlet, so that the total DEGR of system increases. Figure 5 shows the pressure distributions in channel for $\Delta q^{\prime \prime }$ = 0 W·m^−3^. From Figure 5, there is little change in the situation of pressure distribution with changes of airflow Reynolds numbers, but the pressure drop in channel rises with the increases of airflow Reynolds numbers.

### 3.2. Effects of Thermal Conductivity of Heat Source on Heat Dissipation Performance

**Figure 6:**
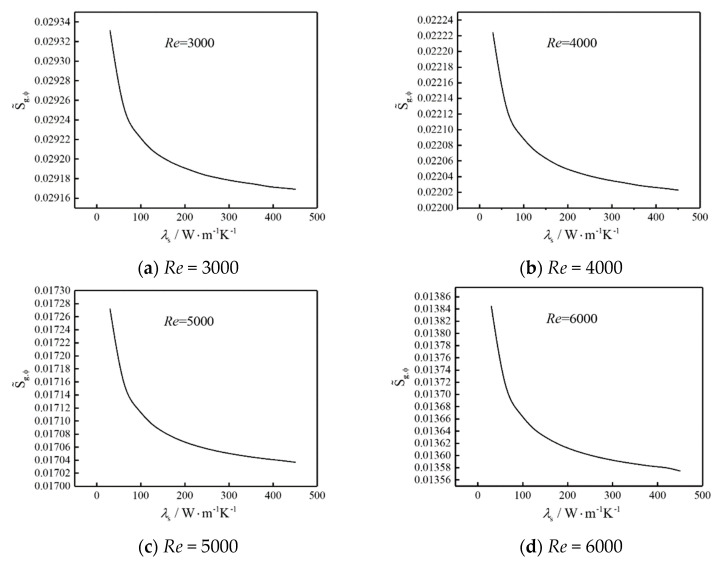
The influence of *λ*_s_ on${\tilde{\;S}}_{g,\phi }$.

Figure 6 shows the influences of thermal conductivity (*λ*_s_) of each heat source on total DEGR (${\tilde{\;S}}_{g,\phi }$) of the system under forced convection with fixed equal heat generating intensity of each heat source. As can be seen from the figure, the ${\tilde{\;S}}_{g,\phi }$ decreases directly and then gradually becomes gentle with the increase of *λ*_s_ at the same Reynolds number of airflow. When *λ*_s_ < 150, the decreasing magnitude is larger, when *λ*_s_ rises to a certain value, the temperature gradients of heat sources are relatively uniform because the thermal resistances of heat sources are smaller. Then, the effect becomes little that improving the heat dissipation performance and reducing the ${\tilde{\;S}}_{g,\phi }$ of the system by simultaneously improving the heat conductivities of heat sources. The improvement effect is more obvious when the thermal conductivity of the heat source is lower.

To study the change of DEGR of system by changing the difference of thermal conductivity with the fixed equal heat generating intensity of each heat source, the thermal conductivities of heat sources are set as *λ*_s,1_, *λ*_s,2_, *λ*_s,3_ and *λ*_s,4_, respectively, and distributed with equal difference along flow direction:

(17) $$\lambda_{s,2}-\lambda_{s,1}=\lambda_{s,3}-\lambda_{s,2}=\lambda_{s,4}-\lambda_{s,3}=\Delta \lambda_{s}$$

where Δ*λ*_s_ is the difference of thermal conductivity between adjacent heat sources.

Considering the sum of thermal conductivities and the constraints for practical thermal conductivities of materials, it is assumed that the thermal conductivities of heat sources satisfy 30 ≤ *λ*_s,I_ ≤ 450 (i = 1, 2, 3, 4).

(18) $$\lambda_{s,\;j}=240+\left(i-2.5\right)\Delta \lambda_{s}\;(-140\leq \Delta \lambda_{s,\;j}\leq 140)$$

**Figure 7:**
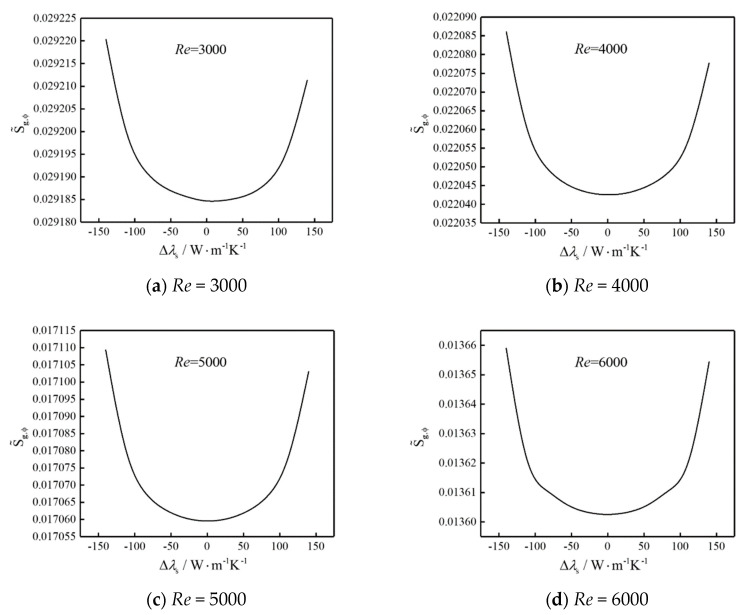
The influences of Δ*λ*_s_ on ${\tilde{\;S}}_{g,\phi }$.

Figure 7 shows the influences of thermal conductivity difference (Δ*λ*_s_) on total DEGR (${\tilde{\;S}}_{g,\phi }$) of system when the heat generating intensities are constants. The results show that the total ${\tilde{\;S}}_{g,\phi }$ of the system decreases first and then rises with the rise of Δ*λ*_s_ and ${\tilde{\;S}}_{g,\phi }$ at Δ*λ*_s_ = −140 W·m^−^^1^·K^−1^ is larger than that at Δ*λ*_s_ = 140 W·m^−^^1^·K^−1^, which means that the thermal conductivity is preferably distributed from small to large in the airflow direction, the device with higher thermal conductivity should be arranged near the channel outlet. Further numerical results listed in Table 3 show that (Δ*λ*_s_)_opt_ for the same optimization objective are different under different fluid flow conditions. From Table 3, when the *Re* of airflow are 2500 and 3000, the optimal difference of thermal conductivity ((Δ*λ*_s_)_opt_ > 0) makes the total ${\tilde{\;S}}_{g,\phi }$ reach minimums; when *Re* of airflow is 4000, 5000 and 6000, the optimal difference of thermal conductivity ((Δ*λ*_s_)_opt_ = 0 W·m^−^^1^·K^−1^) makes the total ${\tilde{\;S}}_{g,\phi }$ reach minimums. 

This occurs because the discrete elliptical cylinders with heat generating reinforce the flow resistance in the channel but cause secondary vortexes at the same time. When the *Re* of airflow is 3000 and Δ*λ*_s_ is larger than 0, the thermal conductivity of heat source increases one by one appropriately to reduce the thermal resistance for thermal conduction in corresponding regions along flow direction, and the temperature gradients decrease obviously resulted from the secondary vortexes which enhance heat transfer locally, so the cooling requirements of all parts can be met better and appropriately, and the total DEGR of system decreases. With the *Re* of airflow increasing, the forced convective condition changes, so the heat transfer including heat conduction and convective heat transfer should be adapted coordinately to benefit heat flow for EGM and the total ${\tilde{\;S}}_{g,\phi }$ of system are the smallest when the thermal conductivities of heat sources are equal.

From Figure 2, Figure 6 and Figure 7, the impacts of distributions about heat generating intensity and thermal conductivity is 6% and 1% roughly. The higher the Reynolds number, the smaller the maximum temperature (*T*_max_) of electrical device. At the same *Re*, as the $\Delta q^{\prime \prime }$ and Δ*λ*_s_ increase, the *T*_max_ decreases firstly and then increases, the Δ*q”*_opt_ and the (Δ*λ*_s_)_opt_ make *T*_max_ minimum, and the Δ*q”*_opt_ and (Δ*λ*_s_)_opt_ are different, respectively, for different performance indicators. 

## 4. Conclusions

In this work, a 3-D heat dissipation model of discrete elliptical cylinders with heat generation on a thermal conduction pedestal with forced convection is established. The effects of heat generation intensity distribution and thermal conductivity distribution on the total DEGR of system are investigated, respectively. The results bring to light that when the total heat generating intensity of heat sources is specified, there is an optimal distribution of the heat generation intensity that makes the total DEGR smallest. The minimums of total DEGR corresponding to optimal differences of heat generating intensity are different at different airflow velocities. The heat generating intensity can be decreased one by one appropriately in flow direction to achieve the best effect.

When the thermal conductivity of each heat source is equal, the total DEGR of system decreases with the simultaneous rise of the thermal conductivity of each heat source and then gradually becomes equilibrium. The thermal conductivity of each heat source can be appropriately increased to enhance heat dissipation. 

When the sum of thermal conductivities of heat sources is fixed, there are optimal distributions of thermal conductivity that minimize the DEGR of system. When the Reynolds number of airflow is smaller, the thermal conductivity of heat source can be increased one by one appropriately in flow direction to achieve the best effect; while the Reynolds number of airflow is larger, the thermal conductivity of each heat source should be equalized to achieve the best effect.

## Figures and Tables

**Table 1:** The influences of $\tilde{H}$ and *b* on $\tilde{T}$.

$\tilde{H}$	0.1	0.2
b	0.4652	0.4835
$\tilde{T}$ [82]	7.2339	13.6509
$\tilde{T}$ of this work	7.1805	13.8243

**Table 2:** The optimal distributions of heat generating intensity for different *Re.*

*Re*	3000	4000	5000	6000
$\Delta q_{\mathrm{opt}}^{\prime \prime }\;$/10^6^ W·m^−3^	−0.6	−0.9	−1.0	−1.1
${\tilde{\;S}}_{g,\phi }$	0.02919	0.02201	0.01702	0.01355

**Table 3:** The optimal distributions of the thermal conductivity for different *Re.*

Re	2500	3000	3500	4000	5000	6000
(Δλ_s_)_opt_ / W·m^−1^·K^−1^	4	6	0	0	0	0
${\tilde{\;S}}_{g,\phi }$	0.03382	0.02919	0.02530	0.02201	0.01702	0.01355

## Nomenclature

*b*	Ratio
*C*_ε1_, *C*_ε2_, *C*_μ_	Empirical constants
*c*	Constant pressure specific heat capacity, J·kg^−1^·K^−1^
***F***	Volume force vector, N
*H*	Thickness of pedestal, m
*H_s_*	Height of heat source, m
$\tilde{H}$	Dimensionless height
**I**	Unit matrix
*k*	Turbulent kinetic energy, J
*L*	Length of pedestal, m
*L_a_*	Short axis of ellipse, m
*L_b_*	Long axis of ellipse, m
*P_k_*	Generation term of turbulent kinetic energy
*P_t_*	The total heating power of heat sources
*Q*	Heat source item including viscous dissipation and pressure work, W·m^−3^
***q***	Heat flux vector, W·m^−2^
*q* ^″^	Heat generating intensity, W·m^−3^
$S_{g,\phi }$	Entropy generation rate, W·K^−1^
${\tilde{\;S}}_{g,\phi }$	Dimensionless entropy generation rate
T	Matrix transpose operator symbol
*T*	Temperature, K
$\tilde{T}$	Dimensionless hot spot temperature
***U***	Velocity vector, m/s
W	Width of pedestal, m

## Greek Symbols

*ε*	Turbulent dissipation rate, %
*λ*	Thermal conductivity, W·m^−1^·K^−1^
*μ*	Dynamic viscosity coefficient, Pa·s
*μ*_T_	Turbulent viscosity coefficient, Pa·s
*ρ*	Density, kg·m^−3^
*σ*_k_, *σ*_ε_	Empirical constants

## Subscripts

b	Heat conductive pedestal
f	Fluid
i	Number of heat source
in	Inlet
opt	Optimum
s	Heat source

## Abbreviations

EGM	Entropy generation minimization
EGR	Entropy generation rate
DEGR	dimensionless entropy generation rate
